# Perspectives of Rare Disease Social Media Group Participants on Engaging With Genetic Counselors: Mixed Methods Study

**DOI:** 10.2196/42084

**Published:** 2022-12-21

**Authors:** Megan Yabumoto, Emily Miller, Anoushka Rao, Holly K Tabor, Kelly E Ormond, Meghan C Halley

**Affiliations:** 1 Department of Genetics Stanford University School of Medicine Stanford, CA United States; 2 Stanford Center for Biomedical Ethics Stanford University School of Medicine Stanford, CA United States; 3 Department of Medicine Stanford University School of Medicine Stanford, CA United States; 4 Health Ethics and Policy Lab Department of Health Sciences and Technology Swiss Federal Institute of Technology (Eidgenössische Technische Hochschule Zurich) Zurich Switzerland

**Keywords:** social media, rare disease, genetic counseling, genetics, genomics, delivery of health care

## Abstract

**Background:**

Social media provides a potential avenue for genetic counselors to address gaps in access to reliable genetics information for rare disease communities. However, only limited research has examined patient and family attitudes toward engaging with genetic counselors through social media.

**Objective:**

Our study assessed the attitudes of members of rare disease social media groups toward engaging with genetic counselors through social media, characteristics associated with greater interest, and the benefits and potential pitfalls of various approaches to such engagement.

**Methods:**

We conducted a mixed methods survey of patients and family members recruited from a systematic sample of rare disease Facebook groups. Patient characteristics and their associations with interest in engagement with genetic counselors were evaluated using univariate and bivariate statistics. Responses to open-ended questions were analyzed using thematic content analysis.

**Results:**

In total, 1053 individuals from 103 rare disease groups participated. The median overall interest in engaging with genetic counselors on social media was moderately high at 7.0 (IQR 4.0-9.0, range 0-10). No past experience with a genetic counselor was associated with greater interest in engaging with one through social media (µ=6.5 vs 6.0, *P*=.04). Participants expressed greatest interest (median 9.0, IQR 5.0-10.0) in engagement models allowing direct communication with genetic counselors, which was corroborated by the majority (n=399, 61.3%) of individuals who responded to open-ended questions explicitly stating their interest in 1-on-1 interactions. When asked what forms of support they would request from genetic counselors through social media, participants desired individualized support and information about how to access services. However, participants also expressed concerns regarding privacy and confidentiality.

**Conclusions:**

Patients and family members in rare disease social media groups appear interested in engaging with genetic counselors through social media, particularly for individualized support. This form of engagement on social media is not meant to replace the current structure and content of genetic counseling (GC) services, but genetic counselors could more actively use social media as a communication tool to address gaps in knowledge and awareness about genetics services and gaps in accessible patient information. Although encouraging, concerns regarding privacy and feasibility require further consideration, pointing to the need for professional guidelines in this area.

## Introduction

Over 10,000 different rare diseases collectively affect an estimated 300 million people worldwide [1]. Approximately 70% of these rare diseases are genetic in etiology, and 80% have symptoms that emerge in childhood [2]. Patients with rare diseases typically experience long delays in diagnosis due to providers’ lack of familiarity with these conditions and limited access to diagnostic testing [3]. Although genomic technologies, such as exome and genome sequencing, continue to identify both new and existing rare genetic disorders, these advances have rapidly outpaced the availability of genetic testing (GT) services [4,5]. Additionally, even after diagnosis, patients report significant challenges in accessing reliable and patient-friendly information about their disease, including prognosis, natural history, and management [6]. As the field of genomics continues to expand, new strategies will be needed to increase access to genetics services for the large and heterogenous population of patients with rare diseases.

Genetic counselors are particularly well positioned to address the informational, social, and emotional needs of patients with rare genetic diseases and their family members [7,8]. Trained in both the clinical implications of genetics and patient- and family-centered communication, genetic counselors are in an ideal position to disseminate accessible information about rare genetic diseases to patients, families, and patient communities [9,10]. However, limited availability of genetic counselors is an ongoing challenge. Although there are an estimated 7000 genetic counselors currently practicing worldwide, over 60% of these counselors practice in North America [11]. Even within the United States, the demand for genetic counselors is placing immense pressure on the workforce [12,13]. Although the field is working to train new genetic counselors to meet the rapidly growing global need, responding to this demand will also require creative and efficient service delivery models [14].

One potential strategy for disseminating information to large numbers of genetics patients on a global scale is through social media [7]. Social media provides an accessible tool for individuals to connect with one another and share information and support, including around health and illness [15-17]. Individuals impacted by rare diseases and their family members are particularly active on these platforms for multiple reasons, including for social and emotional support from those experiencing similar conditions worldwide and to fill in gaps in information about their rare disease due to local providers’ limited exposure to their condition or limited available research in general [10,18,19]. Further, a recent study suggested that patients and family members may be interested in using social media to receive general information about GC and genetics services. However, they also suggest concerns about maintaining privacy and confidentiality in the group environment [9]. Additional information is needed to understand attitudes toward engaging with genetic counselors through social media in the broader rare disease community, how to structure such interactions to balance patient preferences regarding privacy and access, and who might benefit from interactions with genetic counselors on social media platforms.

To address this gap, we conducted a survey of patients with rare genetic diseases and their family members using a systematic sampling structure to include a broad range of rare diseases. We intentionally focused recruitment on current social media users to better understand the benefits and barriers specific to genetic counselor interactions in this context. Here, we report our findings on participants’ attitudes toward engaging with genetic counselors through social media, individual characteristics associated with greater interest in engagement with genetic counselors, and the perceived benefits and drawbacks of various approaches to engaging with genetic counselors in this context.

## Methods

### Study Design

We conducted an online survey from October to December 2021 of patients with rare diseases and their family members participating in social media support groups identified from a systematic sample of rare diseases.

### Ethical Considerations

All study procedures were approved by the Stanford University School of Medicine Institutional Review Board (IRB protocol no. 61783).

### Sampling and Participant Recruitment

Studies of patients with rare diseases often include only a small subset of the more common rare diseases (eg, cystic fibrosis, amyotrophic lateral sclerosis, and Huntington disease) [20]. To address this limitation of the current literature, we used a systematic approach to identifying and recruiting participants from a broad range of rare diseases.

### Identifying Rare Diseases

To recruit patients with rare diseases and their family members, we selected a random sample of rare diseases from the Orphanet database, stratified by disease prevalence [21,22]. Nearly 85% of rare diseases listed in Orphanet are “ultra-rare” (defined as having a prevalence of <1 in 1,000,000), but an estimated 80% of the population burden of rare diseases is attributable to only 4% of rare diseases that are more “common-rare” diseases (defined as having a prevalence of 1-9 in 1,000,000 or greater). To ensure inclusion of both common-rare and ultra-rare diseases, we oversampled for common-rare diseases from Orphanet. Additional parameters were based on estimates that approximately 70% of Orphanet diseases are genetic in etiology and that 30% of ultra-rare diseases and 70% of common-rare diseases are expected to have a Facebook group [2,23]. Based on these estimates, we selected a stratified random sample of 1200 rare diseases with the expectation of identifying a Facebook group for at least 400 different rare diseases and enrolling participants from 100 of the identified groups. After selecting this sample of 1200 rare diseases, we screened each disease on rare disease databases to only include rare diseases with a known or suspected genetic etiology based on information provided by organizations, such as the National Organization of Rare Disorders [24] and GeneReviews [25].

### Identifying Social Media Groups

To identify social media groups for our list of rare genetic diseases, we used Facebook, the largest social media platform available and on which rare disease groups are known to be active [23]. Using a dedicated, study-specific Facebook account for the study’s principal investigator (author MCH), we searched each identified disease in our sample using both the disease’s primary name and up to 5 alternative names listed in Orphanet. Eligibility criteria for groups included (1) categorized as a group on Facebook and (2) explicitly focused on an eligible rare disease per the public group description. If more than 1 group was identified for a single disease, only the group with the largest number of members was included. For ultra-rare diseases with multiple subtypes, umbrella groups covering more than 1 subtype were reviewed and included if the specific subtype was named in the group description (eg, autosomal dominant optic atrophy [ADOA] as the umbrella group for the diseases ADOA Kjer-type and ADOA-plus type).

### Participants and Procedures

To recruit participants, a member of the study team contacted up to 3 moderators or administrators of each identified Facebook group via private message. If moderators and administrators agreed to post the survey link to the group, they were provided with the IRB-approved recruitment language and survey link to share with their group members. We attempted to contact each Facebook group up to 3 times over a 6-week period, and all groups that agreed to post the survey had access to an active survey link for a minimum of 3 weeks. Participant eligibility criteria included (1) aged 18 years or older, (2) able to read and write in English, and (3) self-identified as either a patient with a rare disease or a family member of a patient with a rare disease. All included groups were associated with a disease with a known genetic component. However, a subset of individuals with a recognized genetic disease may be diagnosed clinically, without a molecular diagnosis. All patients and their family members within the identified groups were eligible for participation regardless of whether they had a confirmed molecular diagnosis.

### Measures

The survey instrument was developed through an iterative process. Structured questions were drawn from previously published studies whenever possible and included additional measures not represented in the analysis later (see Multimedia Appendix 1). All new items were developed based on the existing literature [9,10] and were pretested with patients with rare diseases prior to dissemination. The measures in the analysis included (1) sociodemographic characteristics [26,27], (2) self-reported interest in engaging with genetic counselors through social media [9], (3) prior access to and experience with GC and GT [9,28,29], and (4) frequency of social media use and self-reported perceived social connectedness [30]. In addition, we presented 4 proposed models for how engagement with genetic counselors through social media could be structured and asked participants to rate their interest in each on a scale of 0-10, with 10 indicating maximum interest. These models were developed to explore participants’ attitudes toward varying approaches to engagement with increasing levels of direct access to a genetic counselor. The model “minimal engagement” involved the genetic counselor sending information and resources through the moderator, with no direct interaction with other group members. The model “moderate engagement” involved bidirectional communication between the genetic counselor and the moderator only. “Enhanced moderate engagement” involved the genetic counselor joining the group directly but providing only information and resources. Finally, “maximum engagement” involved the genetic counselor joining the group and engaging in bidirectional communication with all group members. Four open-ended questions also were included to elicit participant perspectives on the benefits and drawbacks of these different models. The survey was distributed using Qualtrics software (Qualtrics).

Finally, data on rare disease social media group characteristics were extracted from publicly available Facebook information (eg, size of group, activity within group), and additional data on each rare disease represented in the final sample were extracted from Orphanet (eg, disease classification, inheritance pattern, age of onset). We integrated the social media and rare disease characteristics into individual participant-level data.

### Data Analysis

We used R version 4.1.1 (R Core Team and the R Foundation for Statistical Computing) [31] for quantitative analyses and Microsoft Excel (Microsoft Corporation) for qualitative analysis. Descriptive analyses were performed for all variables using means and SDs to describe normally distributed variables and medians with IQRs for all nonnormally distributed variables. Participants’ reported connectedness to their social media group was summarized as a mean social media connectedness score based on 3 questions representing how connected participants felt to their rare disease group on a 5-point Likert scale from 1 (strongly disagree) to 5 (strongly agree).

Our primary comparative analysis was designed to test a series of hypotheses to investigate whether certain participant, social media group, and rare disease characteristics were associated with overall interest in engaging online (primary outcome variable). To do this, we first performed bivariate analyses (Welch 2-sample *t* tests and ANOVA) to examine the relationship between each hypothesized predictor and the primary outcome variable. We planned to conduct a multivariable analysis (linear regression) if more than 1 independent variable was associated with the primary outcome variable with *P*<.10. However, bivariate analyses resulted in only 1 potential predictor of increased interest in engagement, so we did not perform the planned multivariate analysis.

Responses to open-ended survey questions were analyzed using a thematic content analysis approach. Two team members reviewed the data and developed a draft codebook based on themes the team determined to be most prevalent in the data. We conducted multiple rounds of codebook revision to ensure high interrater reliability (>90% agreement) and then applied codes to the full data set (Supplementary Table 1 in Multimedia Appendix 2) [32]. We calculated frequencies for each code by question and identified exemplary quotes for each code.

## Results

### Social Media Group Member Characteristics

**Figure 1:**
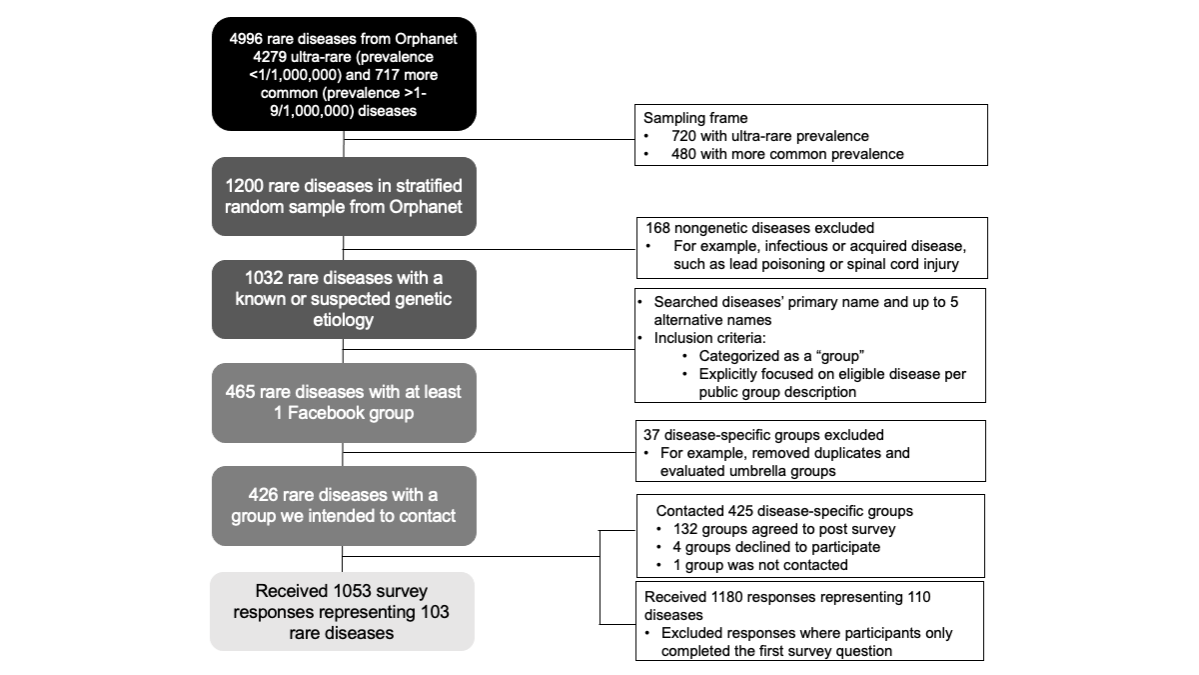
Creation of a systematic random sample of rare diseases included in this study. Note: One group was not contacted because the term “rare disease” was inconsistent with how members of the community identified, as described on the Facebook group’s public description.

A total of 1053 eligible individuals from 103 Facebook groups responded and completed at least 1 survey question following screening (Figure 1). Of note, our final sample included participants with common-rare diseases at rates proportional to population estimates (n=820, 77.8% in our sample vs 80% population estimate). Over half (n=660, 62.7%) of participants self-identified as an adult patient with a rare disease and 37.3% (n=393) as the family member of a patient with rare diseases. Participants had a median age of 43 years (IQR 35-52) and were predominantly non-Hispanic (n=982, 93.3%), White (n=957, 90.9%), and female (n=868, 82.4%). Approximately one-quarter (n=287, 27.3%) of participants lived outside the United States. Additional individual participant characteristics are provided in Table 1 and Supplementary Table 2 in Multimedia Appendix 2; additional participant, rare disease, and social media group characteristics are provided in Supplementary Table 1 in Multimedia Appendix 2.

**Table 1:** Select social media group member demographics (N=1035).

Characteristics		Participants
**Group, n (%)**		
	Patient	660 (62.7)
	Family member	393 (37.3)
Age (years), median (IQR)		43 (35-52)
**Gender, n (%)**		
	Female	868 (82.4)
	Male	153 (14.5)
	Other	13 (1.3)
	Missing	19 (1.8)
**Hispanic^a^, n (%)**		
	No	982 (93.3)
	Yes	59 (5.6)
	Missing	12 (1.1)
**Race^a^, n (%)**		
	White	957 (90.9)
	Black or African American	28 (2.7)
	Asian or Asian American	60 (5.7)
	American Indian or Alaskan Native	18 (1.7)
	Native Hawaiian or other Pacific Islander	2 (0.2)
	Some other race	27 (2.6)
	Missing	15 (1.4)
**Location, n (%)**		
	United States	615 (58.4)
	Outside the United States	287 (27.3)
	Missing	151 (14.3)
**Highest level of education, n (%)**		
	Less than high school	11 (1.0)
	High school or General Educational Development (GED)	129 (12.3)
	Some college or associate degree	265 (25.2)
	Bachelor’s degree	300 (28.5)
	Advanced or graduate-level coursework or degree	299 (28.4)
	Missing	49 (4.7)
**Household income^b^ (US $), n (%)**		
	≤25,000	80 (7.6)
	25,001-50,000	170 (16.1)
	50,001-100,000	252 (23.9)
	100,001-200,000	388 (36.8)
	Prefer not to say/don’t know	114 (10.8)
	Missing	49 (4.7)
**Disease prevalence^b^, n (%)**		
	Unknown	155 (14.8)
	<1 in 1,000,000	78 (7.4)
	1-9 in 1,000,000	172 (16.3)
	1-9 in 100,000	418 (39.7)
	1-9 in 10,000	217 (20.6)
	>1 in 1000	13 (1.2)
**Facebook group disease specification, n (%)**		
	Specific-to-rare disease	1021 (97.0)
	Umbrella rare disease	32 (3.0)
Size of group, median (IQR)^b^		1400 (765-2800)
Number of new posts per month, median (IQR)^b^		36 (19-120)
Number of new members per week, median (IQR)^b^		4 (1-9)

^a^Participants can select more than 1 response.

^b^Information about the rare disease and social media group was integrated into the participant-level data.

The rare diseases represented in our final sample included 17 distinct disease classifications and varied widely in the reported age of onset and inheritance pattern (Supplementary Table 3 in Multimedia Appendix 2). Social media groups included had a median group size of 1400 members (IQR 765-2800), 36 new posts per month (IQR 19-120), and 4 new members per week (IQR 1-9). Additional data on rare diseases and social media groups at both the individual participant and group levels are provided in Supplementary Tables 2 and 3 in Multimedia Appendix 2, respectively.

### Access and Experience with Genetic Counseling and Testing

**Figure 2:**
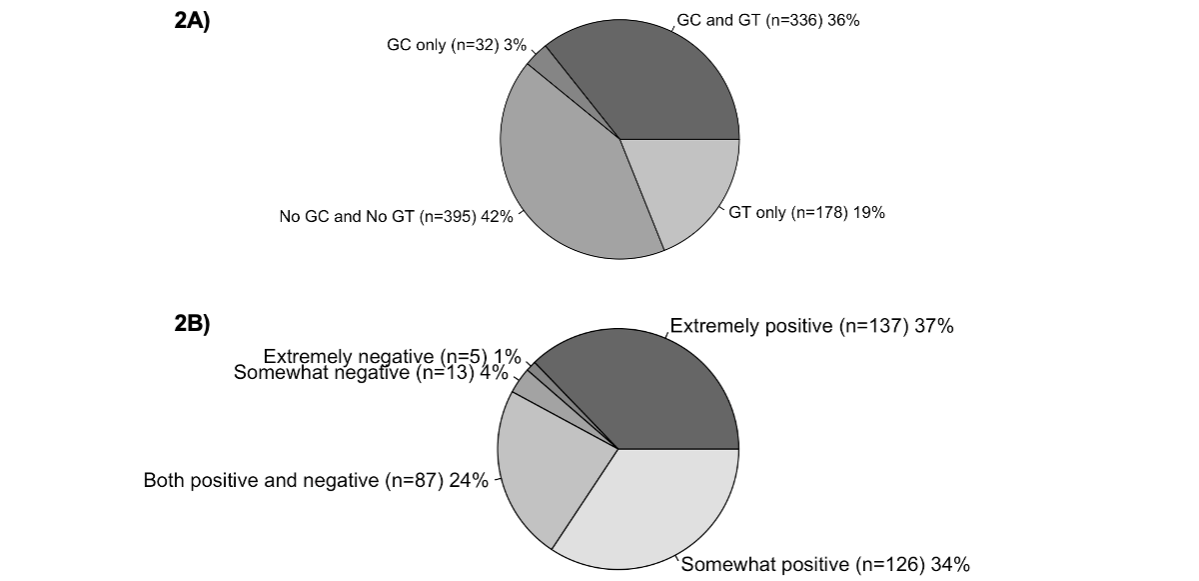
Prior experience with GC and GT. (A) Responses to questions about prior experience with GC or GT services (n=941, 89.4%). The excluded participants either chose not to respond or only responded to 1 question and not the other. (B) Respondents who indicated that they have met with a genetic counselor in the past (n=368, 34.9%) were prompted to describe their previous interactions with genetic counselors on a 5-point Likert scale. GC: genetic counseling; GT: genetic testing.

Participants varied widely in their previous access to GC and GT (Figure 2). Across the sample, 35.7% (n=336) of the participants reported receiving both GC and GT prior to the study, with an additional 18.9% (n=178) reporting only GT and 3.4% (n=32) only GC. The remaining 42.0% (n=396) of the participants had neither met with a genetic counselor nor received GT in the past. Among those who met with a genetic counselor in the past (n=368, 34.9%), the majority reported having somewhat or extremely positive experiences (n=263, 71.5%). The proportion of participants who reported knowing the specific genetic variant that caused their rare disease (n=422, 40.1%) was similar to those without a molecular diagnosis (n=429, 40.7%); see Supplementary Table 2 in Multimedia Appendix 2. The remaining participants reported having only a partial diagnosis or variants of uncertain significance (n=69, 6.6%), designated “other” on the survey item and elaborated further (n=34, 3.2%), or did not respond (n=99, 9.4%).

### Interest in Engaging with Genetic Counselors Online

Participants reported moderately high connectedness with their Facebook support group, with a mean social media connectedness score of 3.74 out of 5 (SD 0.83); see Table 1. Although a subset of participants reported primarily seeking either social and emotional support (n=211, 20%) or informational support related to medical management (n=132, 12.5%), nearly half (n=489, 46.4%) emphasized the equal value of both types of support from social media groups.

**Figure 3:**
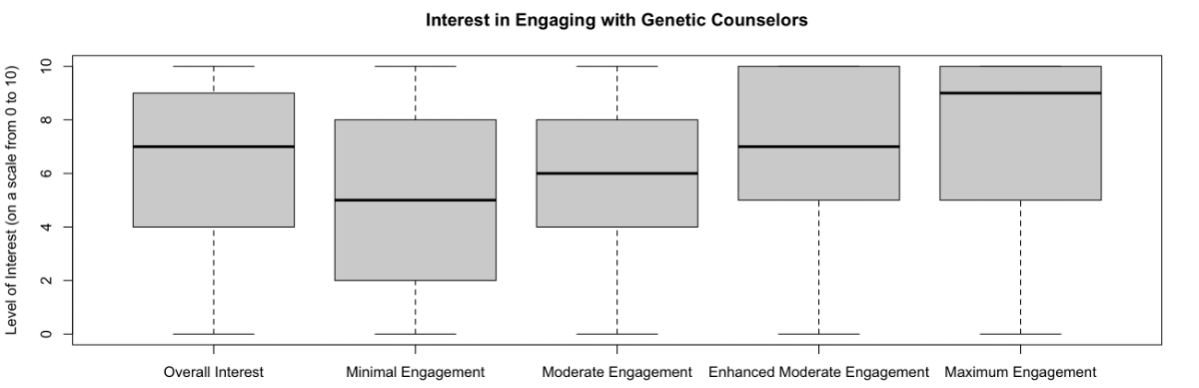
Interest in engaging with genetic counselors. Respondents were asked to indicate how interested they were in engaging with genetic counselors overall and in varying levels of engagement on an 11-point scale from 0 (not at all interested) to 10 (extremely interested). Overall interest: I am interested in interacting with a genetic counselor on social media. Minimal engagement: The genetic counselor sends information and resources to the moderator of the group but is unavailable to answer specific questions. Moderate engagement: The genetic counselor communicates directly with the moderator of the group and addresses questions that the moderator requests the genetic counselor's input on. Enhanced moderate engagement: The genetic counselor is a member of the group and can post information and resources to the group as they see fit. Maximum engagement: The genetic counselor is a member of the group and can answer questions directly from group members.

Overall interest in engaging with genetic counselors on social media was also high, with a median score of 7 out of 10 (IQR 4-9); see Figure 3. When asked for their interest in 4 different models of engagement with varying degrees of access to genetic counselors, the participants’ level of interest increased as the extent of direct access to a genetic counselor increased. This was reflected in a median interest score of 9 out of 10 (IQR 5-10) for the model with maximum engagement compared to a score of 5 out of 10 (IQR 2-8) for the model with the most minimal engagement.

Bivariate analysis results of individual participant, group, and rare disease characteristics hypothesized to correlate with higher interest in engaging on social media are summarized in Supplementary Table 4 in Multimedia Appendix 2. Most notably, participants who had not previously met with a genetic counselor expressed greater interest in engaging with genetic counselors online (µ=6.47) than those who did have prior experience (µ=6.01, t_837_=2.09, *P*=.04). Additionally, participants who lived outside the United States expressed greater interest in engaging with genetic counselors online (µ=6.68) than those who lived within the United States (µ=6.19, t_743_=1.96, *P*=.05). All other associations assessed were not statistically significant (*P*>.05).

### Perceived Benefits and Drawbacks of Engaging with Genetic Counselors Through Social Media

A total of 732 (69.5%) participants contributed a written response to at least 1 open-ended question asking how they wanted to interact with genetic counselors on social media, what they were looking for in these interactions, and why they wanted to engage. When asked about the forms of engagement they most desired, 1-on-1 individual meetings were most preferred (n=399, 61.3%), followed by group-based interactions via social media (n=243, 37.3%); see Table 2. Primary resources the participants hoped to access through engaging with genetic counselors included answers to questions about their specific disease, such as inquiries about the impact of the disease on their family (n=83, 12.5%), available treatment (n=74, 11.1%), and GT (n=123, 18.5%). The primary benefit of social media engagement with genetic counselors was increased accessibility of information (n=189, 28.4%), followed by increased reliability of available information (n=184, 27.7%). The primary concern raised regarding engagement with genetic counselors on social media was the lack of a personal relationship between the patient and the genetic counselor (n=202, 40.2%), with concerns about privacy and confidentiality (n=90, 17.9%) and lack of trust (n=71, 14.1%) also frequently cited. Additional subthemes are illustrated in Table 2.

**Table 2:** Thematic analysis of open-ended questions.

Themes and subthemes		Participants, n (%)	Illustrative quotation
**How do social media group members want to engage with genetic counselors? (n=651)**			
	1-on-1 interactions	399 (61.3)	“Individual sessions, through messages or call (voice or video)” [participant (P)193]
	Group-based interactions	243 (37.3)	“Closed Facebook group or a more secure location for group if possible while being user friendly” [P289]
	Information only	47 (7.2)	“Provide resources but not directly answering questions” [P1067]
	Through moderator	7 (1.1)	“Best is for moderator to pass along information and let members contact GCs if they would like” [P9]
	Do not bother	23 (3.5)	“I don’t, I think it’s a private matter that should be discussed in an office setting” [P466]
**What type of support are social media group members looking for? (n=665)**			
	Available to answer questions	278 (41.8)	“To be able to answer questions when needed” [P1042]
	Available to answer questions: inquiries about family members	83 (12.5)	“Would like info I could share with my offspring and extended (family)” [P293]
	Available to answer questions: inquiries about prognosis	34 (5.1)	“Information about what to expect” [P1146]
	Available to answer questions: inquiries about treatment	74 (11.1)	“Information to get latest treatment options” [P1051]
	Access to services	123 (18.5)	“Logistical support about how and where to get testing done (and how to pay for it)” [P325]
	Research/clinical trials	94 (14.1)	“Connecting us with up to date information on genetic studies about the disease, opportunities to participate in research studies” [P1070]
	Unsure	106 (15.9)	“I'm not sure what a GC knows or has to offer me” [P830]
**What are benefits of engaging with genetic counselors on social media? (n=665)**			
	Accessible/convenient	189 (28.4)	“Benefits would be access to the knowledge or advice easier than waiting for your yearly appointment with the GCs” [P957]
	Reliable information	184 (27.7)	“To have someone who is an expert” [P843]
	Psychosocial support	100 (15.0)	“Better understanding and less anxiety” [P748]
	No benefits	40 (6.0)	“None! We don't need counselors, we need genetic testing!” [P751]
**What are drawbacks of engaging with genetic counselors on social media? (n=503)**			
	Lack of personal relationship	202 (40.2)	“There may be some disconnect between the patient & counselor due to them not being in the same location. It can be difficult to pick up on all the silent communication cues when online - even with video conferencing” [P73]
	No drawbacks	93 (18.5)	“Really can't think of any drawbacks” [P99]
	Privacy/confidentiality	90 (17.9)	“Confidentiality, I enjoy having nonmedicalized spaces to discuss my condition and having a provider there re-medicalizes it” [P128]
	Lack of trust	71 (14.1)	“Lack of trust in someone you can't see face to face, general mistrust of giving info to an unknown internet contact” [P353]
	Irrelevant information	39 (7.8)	“Wrong info could be given for what’s accessible where you live” [P145]
	Frightening information	21 (4.2)	“Fear of what I might learn about my future” [P11]
	Other	26 (5.2)	“Feeling like you don’t need to go to appointments or the doctor because you found info online” [P483] “Too much asked of the counselors” [P444]

## Discussion

### Principal Findings

Our findings from a large survey of patients with rare diseases and their family members across 103 rare disease social media support groups showed high interest in engaging with genetic counselors through social media. Participants who had never met with a genetic counselor in the past expressed greater overall interest in communicating with one on social media than those who had met with a genetic counselor. Moreover, participants preferred models in which genetic counselors were engaged and interactive. Those who elaborated on their interest expressed a desire for 1-on-1, personalized support from a genetic counselor, who they perceived to be a reliable source of information about their rare disease.

Our results suggest higher levels of interest in engaging on social media with genetic counselors than have previously been reported, though this may be attributable to our focus on the perspectives of current social media users [9]. The qualitative data we obtained further reinforced participants’ interest in patient–genetic counselor interactions to find answers to their specific questions from reliable sources. Our findings also highlight patient concerns about privacy and confidentiality that may continue to discourage some from engaging with genetic counselors on social media [7,9,33].

Our findings also provide insights regarding access to GC and GT across rare diseases. Many participants within our cohort received GT but had never met with a genetic counselor to explain the test results or to obtain additional information at the time of results disclosure. This suggests that the global scaling-up of GC services is not occurring fast enough to match the expanding implementation of genetic and genomic technologies in the clinical setting. Given that GC services tend to be delivered less systematically in low- and middle-income countries due to costs [34], this gap in care is likely to disproportionately impact patients with rare diseases who are already underserved within the health care system globally [6].

Our findings also have implications for both clinical practice and policy. Many physicians, health care organizations, and nonprofits are already using social media to disseminate health information directly to patient communities for free or at minimal cost [35,36]. For example, providers in the fields of hematology and oncology use social media to provide medical education, rapidly disseminate new information, and encourage patients to engage in their health care [37,38]. Within the clinical context, a genetic counselor can provide patients with accessible information about a given condition, whenever available, and evaluate a patient’s or family’s response to the information. Participants’ interest in high levels of engagement points to the informational support a genetic counselor may uniquely be able to provide in an online setting. Genetic counselors are often employed by academic medical centers, private and public hospitals, and diagnostic laboratories, and their services are charged to health insurance payers for eligible individuals. The type of support participants requested in their written responses accurately underlined roles and responsibilities that fall within the genetic counselors’ scope of practice (eg, information about recurrence risk, prognosis, treatment, access to GT). However, the extent to which individualized support is desired would likely require more time commitment than a genetic counselor could feasibly provide outside of work hours. Within the context of this particular study, the proposed models of engagement with genetic counselors on social media implied that a genetic counselor would be available to provide support at no cost to the social media support group. However, the requested 1-on-1 interactions are essentially the equivalent of a clinical consultation and may not be sustainable without appropriate compensation for this form of service. There is little legal precedent to inform recommendations for engaging potential patients on social media, though genetic counselors should be aware of state and federal legal requirements in place that may prohibit such engagement [39]. By raising awareness of GC, some individuals may be more able to seek out these clinical services than others, given discrepancies in access by geographic location [11].

Further, genetic counselors engaging with social media groups would need to be careful to avoid providing medical advice outside of their scope of practice. A genetic counselor interested in engaging with these social media groups could potentially manage providing informational support, but genetic counselors are unlikely to be able to provide the higher level of engagement and dynamic dialogue patients and family members desire. Current technological advances, such as artificial intelligence, are being investigated as a potential means for delivering services in both health care broadly and within the field of genomics, which could bolster different approaches to addressing the needs of rare disease communities [40,41]. This may be more achievable from a time and labor standpoint, but the informational gaps may not be as amenable to this type of support as more well-established and researched diagnoses, such as cancer [42].

Although engagement through social media may be able to fill some of the gaps in knowledge that arise when patients obtain a diagnosis but are unable to meet with a genetic counselor, a professional may need to actively contribute to the platform’s knowledge base to effectively fill these gaps. Studies examining the impact of these efforts in other medical specialties suggest the need to establish best-practice guidelines that address both the provider’s motivations and their ability to set boundaries [8,41,42]. Common guidelines for the use of social media by health care providers also highlight the responsibility to only share information from credible sites, refute any inaccurate information encountered, and use the most secure privacy settings available [43]. This points to the practical and logistical concerns that have already arisen in this setting, including balancing providing broad medical information that might help inform decision-making and avoiding the direct provision of medical advice.

Current guidelines, to the extent that they exist within the genetic counselor profession, encourage genetic counselors to be aware of concerns for patient and provider privacy on social media platforms and establish ethical and professional boundaries for themselves. However, there are no guidelines for genetic counselors that demonstrate what this might look like in practice [7,9,10]. Professional organizations, such as the National Society of Genetic Counselors (NSGC) and the American College of Medical Genetics (ACMG), can play a key role in leading the discussion to provide this support. Guidelines will need to include recommendations regarding the types of information to be communicated, the extent of the engagement, and how providers should address ethical concerns that may arise while they are acting as a liaison for the health care system online. It also will be critical to involve patient stakeholders in the creation of these guidelines to determine the best step forward. Although our study suggests high interest in relationships with genetic counselors on social media among the patient and family communities, clearer guidance is needed to address the systems-level issues and concerns genetic counselors may have.

There are clearly many unanswered questions that must be explored in greater depth before patient–genetic counselor interactions through social media are pursued by both interested parties. Further exploration is necessary to consider the goals and outcomes of having a genetic counselor engage with these groups and how success could be measured. Although there is a dire need for alternative approaches to providing patients with rare diseases with reliable sources of information, patient interest alone does not serve as an indication that this is a feasible option for genetics professionals. These interactions are not meant to replace the current structure and content of GC services, but genetic counselors could use social media as a communication tool for addressing gaps in knowledge and awareness about genetics services and gaps in accessible patient information on a global scale.

### Limitations

Our study has several limitations. First, we chose to focus on current social media users, and therefore, our data cannot inform our understanding of perspectives of those who are not currently using social media. Second, we were unable to accurately calculate the response rate because we were unaware of how many people viewed the survey throughout the duration of recruitment. Although we collected the number of members in each group at the time of recruitment, this gives little to no indication of the number of active users of these groups who might have seen the study but opted not to respond. Third, we cannot be certain whether all participants have access to GC services in their respective countries. We did not collect the specific countries in which our participants reside in order to understand who undergoes GC and how these services are used in different countries. It also is possible (or even likely) that participants’ interest in engaging with genetic counselors through social media also reflects their interest in accessing a genetic counselor in any context.

Using social media as a tool for recruitment is also known to result in a lack of gender, ethnic, racial, and socioeconomic diversity, which is reflected in the sociodemographic characteristics of our sample [20,44]. Compared to the population of Facebook users, a greater proportion of participants in our sample identified as White (67% vs 91%) and female (77% vs 82%), while a smaller proportion reported having household incomes over US $75,000 (73% vs 60%) or a college degree (73% vs 57%) [44]. Additional research is needed to ensure inclusion of diverse perspectives, including of those who do not participate in social media support groups. Furthermore, the heterogeneity and lack of systematic characterization of the rare disease community at large also made it difficult to assess the extent to which our sample captured and reflected key points of variation across the rare diseases. A complete analysis of nonresponders should be performed to further investigate the social media group and rare disease characteristics not represented here in this study.

### Conclusion

The results of this study demonstrate that patients with rare diseases and their family members are interested in engaging with genetic counselors on social media as a tool to bridge the current gaps in access to genetics resources. However, the extent to which they desire 1-on-1 interactions raises privacy and confidentiality concerns, as well questions of the scope of practice associated with patient-provider interactions on social media. The data presented in this study therefore illustrate the need for guidelines to facilitate these interactions and to advance the conversation within the genetics community about the use of social media as an opportunity for engagement and information dissemination to meet the variegated, evolving, and complex needs of patients with rare diseases.

## Appendix

Multimedia Appendix 1 Complete survey instrument.

Multimedia Appendix 2 Supplementary tables.

## Abbreviations

ADOA: autosomal dominant optic atrophy
GC: genetic counseling
GT: genetic testing
IRB: Institutional Review Board
