# Supplementary material for: Perspectives of Rare Disease Social Media Group Participants on Engaging With Genetic Counselors: Mixed Methods Study
Source: J Med Internet Res. 2022 Dec 21;24(12):e42084. doi: 10.2196/42084 (PMC9813816; doi:10.2196/42084)
Supplement: Multimedia Appendix 1 [file jmir_v24i12e42084_app1.docx]

**Perspectives of Rare Disease Social Media Group Participants on Engaging with Genetic Counselors: Mixed Methods Survey**

Megan Yabumoto[1], Emily G. Miller[2], Anoushka Rao[2], Holly K. Tabor[2,3] Kelly E. Ormond[1,2,4], and Meghan C. Halley[2]

[1] Department of Genetics, Stanford University School of Medicine, Stanford, CA, USA

[2] Stanford Center for Biomedical Ethics, Stanford University School of Medicine, Stanford, CA, USA

[3] Department of Medicine, Stanford University School of Medicine, Stanford, CA, USA

[4] Health Ethics and Policy Lab, Department of Health Sciences and Technology, Swiss Federal Institute of Technology (Eidgenössische Technische Hochschule-Zurich), Zurich, Switzerland

**Supplemental Material 1: Complete survey instrument**

**DESCRIPTION:** You are invited to participate in a Stanford University survey to better understand the experiences and perspectives of patients and families affected by a broad range of rare diseases. While each individual rare disease is inherently unique, rare disease patients and families also may share similar challenges to accessing healthcare simply because their disease is rare. In this survey, we will ask about your (or your family member’s) personal and health background, your experiences with healthcare access, diagnosis and treatment, as well as your attitudes towards engaging with clinicians and researchers both in-person and online. We will ask for your email address if you are willing to participate in follow-up surveys, though this is not required to participate.

**RISKS AND BENEFITS:** The risks associated with this study are minimal and include loss of confidentiality and psychological distress due to answering questions about challenging health issues. To minimize these risks, your answers will be stored on secured servers at Stanford University, and will not include any information that can identify you. You can skip any questions that you do not want to answer and you can stop taking the survey at any time.

We cannot guarantee or promise that you will receive any benefits from this study. The information gathered through this study is intended to increase our understanding of the challenges faced by the rare disease community.

**TIME INVOLVEMENT:** The survey will take approximately 15 minutes to complete.

**PAYMENTS:** At the end of the survey, you will be given the option to enter a voluntary drawing to win one of twenty Visa gift cards valued at $50 each. If you choose to enter, you will be taken to a separate website and asked for your email address so that we can contact you, should you win. The drawing will be conducted by the Stanford Center for Biomedical Ethics in Palo Alto, California. Participation in the study is not required in order to participate in the raffle. You may enter the raffle if you do not start or complete the study task. The chance of winning a prize is dependent on how many participants enter the raffle. The winner will be notified by email no later than ten days after the survey is closed and provided with information on how to accept and collect the gift card.

**PARTICIPANT’S RIGHTS:** If you have read this form and have decided to participate in this project, please understand your participation is voluntary and you have the right to withdraw your consent or discontinue participation at any time without penalty or loss of benefits to which you are otherwise entitled. The alternative is not to participate. You have the right to refuse to answer particular questions. The results of this research study may be presented at scientific or professional meetings or published in scientific journals. Your individual privacy will be maintained in all published and written data resulting from the study. Identifiers, including email addresses, might be removed from identifiable private information and, after such removal, the information could be used for future research studies or distributed to another investigator for future research studies without additional informed consent from you.

 **CONTACT INFORMATION:** *Questions:* If you have any questions, concerns, or complaints about this research, its procedures, risks and benefits, contact the Protocol Director, Meghan Halley (mhalley@stanford.edu).

*Independent Contact:* If you are not satisfied with how this study is being conducted, or if you have any concerns, complaints, or general questions about the research or your rights as a participant, please contact the Stanford Institutional Review Board (IRB) to speak to someone independent of the research team at 650-723-5244 or toll free at 1-866-680-2906. You can also write to the Stanford IRB, Stanford University, 1705 El Camino Real, Palo Alto, CA 94306.
  
**Authorization To Use Your Health Information For Research Purposes** Because information about you and your health is personal and private, it generally cannot be used in this research study without your authorization. If you agree to participate, it will provide that authorization. The form is intended to inform you about how your health information will be used or disclosed in the study. Your information will only be used in accordance with this authorization form and the informed consent form and as required or allowed by law. Please read it carefully before agreeing to participate. 
 
**What is the purpose of this research study and how will my health information be utilized in the study?** The purpose of this study is to better understand the experiences and perspectives of patients and families affected by a broad range of rare diseases. Your health information will be used to understand the rare or undiagnosed disease you or your family is dealing with and the challenges faced by the rare disease community.

**Do I have to agree to this authorization form?** You do not have to agree to this authorization form. But if you do not, you will not be able to participate in this research study. Agree with this form is not a condition for receiving any medical care outside the study.

**If I agree, can I revoke it or withdraw from the research later?** If you decide to participate, you are free to withdraw your authorization regarding the use and disclosure of your health information (and to discontinue any other participation in the study) at any time. After any revocation, your health information will no longer be used or disclosed in the study, except to the extent that the law allows us to continue using your information (e.g., necessary to maintain integrity of research). If you wish to revoke your authorization for the research use or disclosure of your health information in this study, you must write to: Dr. Meghan Halley, mhalley@stanford.edu.

**What Personal Information Will Be Obtained, Used or Disclosed?** Your health information related to this study, may be used or disclosed in connection with this research study, including, but not limited to, information about your or your family member’s rare or undiagnosed disease. Who May Use or Disclose the Information? The following parties are authorized to use and/or disclose your health information in connection with this research study:
 • The Protocol Director, Dr. Meghan Halley
 • The Stanford University Administrative Panel on Human Subjects in Medical Research and any other unit of Stanford University as necessary
 • Research Staff

**Who May Receive or Use the Information?** The parties listed in the preceding paragraph may disclose your health information to the following persons and organizations for their use in connection with this research study:
 • The Office for Human Research Protections in the U.S. Department of Health and Human Services

Your information may be re-disclosed by the recipients described above, if they are not required by law to protect the privacy of the information. 

**When will my authorization expire?** Your authorization for the use and/or disclosure of your health information will end on September 1, 2051 or when the research project ends, whichever is earlier.
 
Please print a copy of this page for your records. 
 
***If you agree to participate in this research, please click the "next" button below to continue to the survey.***

Are you a citizen of the **European Union** and/or **European Economic Area**? Please click YES for additional information.

Yes

No

**General Data Protection GDPR Consent Form**
As described elsewhere in this informed consent form, during the study, data pertaining to your participation in the study will be generated and recorded. In addition, we will collect from you your personal data and sensitive personal data, including health-related data. We refer to all such data as “questionnaire responses” which will be specifically regulated in the EU/EEA under the General Data Protection Regulation (the “GDPR”). Questionnaire responses may be processed or used for the following purposes, which we refer to, collectively, as “Data Processing”:

 - to carry out the study;
 - to confirm the accuracy of the study;
 - to monitor that the study complies with applicable laws as well as best practices developed by the research community;
 - to make required reports to domestic and foreign regulatory agencies and government officials who have a duty to monitor and oversee studies like this one; and,
 - to comply with legal and regulatory requirements, including requirements that data from this study, without information that could directly identify you, be made available to other researchers not affiliated with the study sponsor or with the study team. It is possible, for example, that as part of efforts to make research data more widely available to researchers, regulatory authorities in some countries may require that Your Study Data, without information that could directly identify you, be made publicly available on the internet or in other ways.

 The following entities and organizations may engage in Data Processing of Your Study Data:
 - the study team, including other people who, and organizations that, assist the study team
 - the ethics committee or institutional review board that approved this study; and
 - domestic and foreign regulatory agencies and government officials who have a duty to monitor or oversee studies like this one.

We may conduct the study in the United States or in other countries where the laws do not protect your privacy to the same extent as the laws in your country of residence. In addition, we may disclose Questionnaire Responses for Data Processing to entities and individuals located in the United States or in other countries where the laws do not protect your privacy to the same extent as the laws in your country of residence. However, all reasonable steps will be taken to protect your privacy in accordance with the applicable data protection laws.

Stanford University takes part in the EU-U.S. Privacy Shield Framework. According to European Commission Implementing Decision (EU) 2016/1250, the EU-U.S. Privacy Shield provides an adequate level of protection for questionnaire responses. (See, https://www.privacyshield.gov/list)

The GDPR gives you certain rights with regard to questionnaire responses. You have the right to request access to, or rectification or erasure of, questionnaire responses. You also have the right to object to or restrict our Data Processing of questionnaire responses. Finally, you have a right to request that we move, copy or transfer questionnaire responses to another organization. In order to make any such requests, please contact mhalley@stanford.edu.

There is no limit on the length of time we will keep questionnaire responses for this research because it may be analyzed for many years. We will also retain questionnaire responses to comply with our legal and regulatory requirements. We will keep it as long as it is useful, unless you decide you no longer want to take part. You are allowing access to this information indefinitely as long as you do not withdraw your consent.

You may withdraw your consent at any time. If you withdraw your consent, this will not affect the lawfulness or our collecting, use and sharing of questionnaire responses up to the point in time that you withdraw your consent. Even if you withdraw your consent, we may still use questionnaire responses that has been anonymized so that the data no longer identifies you. In addition, we may use and share questionnaire responses that has been pseudonymized (by removal of your name and certain other identifiers so that the data does not directly identify you) as permitted by applicable law for purposes of: (a) public health (e.g., ensuring high standards quality and safety of health care and/or of medicinal products or medical devices), (b) scientific or historical research or statistical analysis as permitted by applicable European Union or European Union Member State laws and (c) archiving in the public interest. Further, we will maintain questionnaire responses in fully identifiable form if required by law.

You consent to the collection, use and transfer of questionnaire responses which includes health and other sensitive personal data, for the purpose of carrying out the research study and know that you can withdraw your consent at any time, and we will stop processing your personal data, except as described above.

Please print a copy of this page for your records.

***If you agree to participate in this research, please click the "next" button below to continue to the survey.***

**Instructions:**Please read each of the following questions carefully. The survey will automatically go to the next question when you click your answer. You can click the "back" button to change your answer at any time.

Q1 Which of the following describes you? If more than one applies, **please select one** for the purpose of answering this survey.

I have a diagnosed or suspected (undiagnosed) rare disease myself

I am a family member of someone with a diagnosed or suspected (undiagnosed) rare disease

None of the above

Q2 What **year** were you born?

▼ 2003 ... 1935

Q3 Are you of **Hispanic, Latino, or Spanish** origin? *(check all that apply then click "next")*

No, not of Hispanic, Latino, or Spanish origin

Yes, Mexican, Mexican American, Chicano/a

Yes, Puerto Rican

Yes, Cuban

Yes, another Hispanic, Latino, or Spanish origin (please specify):

Q4 What is your **race**? *(check all that apply)*

White

Black or African American

American Indian or Alaskan Native

Asian Indian

Chinese

Filipino

Japanese

Korean

Vietnamese

Other Asian (Please specify):

Native Hawaiian

Samoan

Chamorro

Other Pacific Islander (Please specify):

Some other race (Please specify):

Q5 With which **gender** do you currently identify?

Female

Male

Transgender male

Transgender female

Non-binary

Prefer to self-describe (please specify):

Prefer not to say

Q6 **Where** do you live?

United States of America

North America (other than USA)

South America

Europe

Australia

Asia

Africa

Other (please specify):

Q7 Which **state** do you live in?

▼ Alabama ... Wyoming

Q8 What type of **community** do you live in?

Large city

Suburb near a large city

Small city or town

Rural area

Q9 What is the highest degree or **level of school** you have completed?

Less than high school

High school or GED

Some college or associate's degree

Bachelor's degree

Advanced or graduate level coursework or degree

Q10 Are you **currently covered** by any of the following forms of **insurance**? *(check all that apply)*

Medicaid, CHIP, or other state-based program

Medicare

Employer-sponsored private health insurance

Other private health insurance

Other health insurance (please specify):

I do not have any form of health insurance.

Q11 Are you currently **employed**?

Yes, full time

Yes, part time

Not currently employed

Q12 How many people are currently living in your household? **Include yourself and all adults and children** for whom your home is their permanent residence regardless of their relationship to you.

▼ 1 ... 15+

Q13 What is your best estimate of the **total income of all family members in your household**, before taxes, in the last year?

Less than $25,000

$25,001-$50,000

$50,001-$100,000

$100,001-$200,000

More than $200,001

Prefer not to say

Don't know/not sure

Q14 Do you identify as having a **disability**?

Yes

No

**Genetic testing looks for changes in your DNA**, sometimes called mutations or variants. For example, it can **provide a diagnosis** for a genetic condition **or inform you about your risk** to develop cancer. This could include tests such as single gene, multigene panel, chromosome microarray, exome sequencing, whole genome sequencing, amniocentesis etc.

Q15 Have you had any **genetic testing** in the past?

Yes

No

Don't know/not sure

Q16 How old were you when you first **started experiencing symptoms** of your rare disease?

___ years old (enter a whole number; if you were less than 1, enter 0)

Before I was born / "in utero"

Don't know, not sure

Q17 How old were you when you were **officially diagnosed** with your rare disease?

___ years old (enter a whole number; if you were less than 1, enter 0)

Before I was born / "in utero"

I don't have an official diagnosis for my rare disease

Don't know/not sure

Q18 Do you have a genetic diagnosis? A genetic diagnosis is **an identified genetic mutation** that causes your rare disease.

Yes, a genetic test has **identified the variant** that causes my rare disease.

I have a **partial genetic diagnosis** that explains some, but not all, of my symptoms.

I have **variants of unknown significance** that may or may not cause my rare disease.

No, **I do not know** the genetic cause of my rare disease.

Other (please specify):

We would like to form an impression of your caregiving situation.

Please select a response to indicate **which description best fits your caregiving situation at the moment.** Please tick only one box per description: ‘no’, ‘some’ or ‘a lot of ’

Q19 I have ____________ fulfillment from carrying out my care tasks.

No

Some

A lot of

Q20 I have ____________ relational problems with the care receiver (e.g., he/she is very demanding or he/she behaves differently; we have communication problems).

No

Some

A lot of

Q21 I have ____________ problems with my own mental health (e.g., stress, fear, gloominess, depression, concern about the future).

No

Some

A lot of

Q22 I have ____________ problems combining my care tasks with my daily activities (e.g., household activities, work, study, family and leisure activities).

No

Some

A lot of

Q23 I have ____________ financial problems because of my care tasks.

No

Some

A lot of

Q24 I have ____________ support with carrying out my care tasks, when I need it (e.g., from family, friends, neighbors, acquaintances).

No

Some

A lot of

Q25 I have ____________ problems with my own physical health (e.g., more often sick, tiredness, physical stress).

No

Some

A lot of

Q26 How **happy** do you feel at the moment?
 
This scale is numbered from 0 to 10. 10 means completely happy. 0 means completely unhappy.

|  | completely unhappy | | | | | | completely happy | | | | | |
| --- | --- | --- | --- | --- | --- | --- | --- | --- | --- | --- | --- | --- |
|  | 0 | 1 | 2 | 3 | 4 | 5 | | 6 | 7 | 8 | 9 | 10 |
| Please move the slider to indicate how happy you feel at the moment | 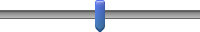 | | | | | | | | | | | |

Under each heading, please check the ONE box that best describes your health TODAY.

Q27 **Mobility**

I have no problems in walking about

I have slight problems in walking about

I have moderate problems in walking about

I have severe problems in walking about

I am unable to walk about

Q28 **Self-Care**

I have no problems washing or dressing myself

I have slight problems washing or dressing myself

I have moderate problems washing or dressing myself

I have severe problems washing or dressing myself

I am unable to wash or dress myself

Q29 **Usual Activities**

I have no problems doing my usual activities

I have slight problems doing my usual activities

I have moderate problems doing my usual activities

I have severe problems doing my usual activities

I am unable to do my usual activities

Q30 **Pain/Discomfort**

I have no problems pain or discomfort

I have slight pain or discomfort

I have moderate pain or discomfort

I have severe pain or discomfort

I have extreme pain or discomfort

Q31 **Anxiety/Depression**

I am not anxious or depressed

I am slightly anxious or depressed

I am moderately anxious or depressed

I am severely anxious or depressed

I am extremely anxious or depressed

Q32 We would like to know how good or bad your health is **TODAY**. 
 
This scale is numbered from 0 to 100. 
 
100 means the best health you can imagine. 0 means the worst health you can imagine.

|  | The worst health you can imagine | | | | | | The best health you can imagine | | | | | |
| --- | --- | --- | --- | --- | --- | --- | --- | --- | --- | --- | --- | --- |
|  | 0 | 10 | 20 | 30 | 40 | 50 | | 60 | 70 | 80 | 90 | 100 |
| Please move the slider to indicate how your health is TODAY | 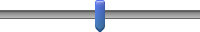 | | | | | | | | | | | |

Genetic counselors are healthcare professionals who **give you information about how genetic conditions** might affect you or your family. They collect personal and family health history to determine how likely it is that you or your family members have a genetic condition. They can **help you decide if genetic testing** is right for you, and **explain the results of a genetic test**.

Q33 Have you ever **met with** **a genetic counselor** in the past?

Yes

No

Don't know/not sure

Q34 Overall, how would you **describe your interaction(s)** with the genetic counselor(s) in the past?

extremely negative

somewhat negative

both positive and negative

somewhat positive

extremely positive

The following questions will ask you about your interest in **engaging with genetic counselors online.**

Q35 Please indicate **how much you agree** with the following statement on a scale from 0 (strongly disagree) to 10 (strongly agree).

|  | strongly disagree | | | | | | strongly agree | | | | | |
| --- | --- | --- | --- | --- | --- | --- | --- | --- | --- | --- | --- | --- |
|  | 0 | 1 | 2 | 3 | 4 | 5 | | 6 | 7 | 8 | 9 | 10 |
| I am interested in **interacting with a genetic counselor** on social media, if available. | 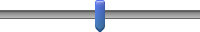 | | | | | | | | | | | |

Q36 Please indicate **how interested you are** in each of the following **types of engagement** with genetic counselors on social media on a scale from 0 (not at all interested) to 10 (extremely interested).

|  | not at all interested | | | | | | extremely interested | | | | | |
| --- | --- | --- | --- | --- | --- | --- | --- | --- | --- | --- | --- | --- |
|  | 0 | 1 | 2 | 3 | 4 | 5 | | 6 | 7 | 8 | 9 | 10 |

| **Minimal engagement:** for example, The genetic counselor **sends** information, resources, and opportunities for the group to the moderator of the Facebook group, **but is unavailable** to address specific supportive care needs of group members. | 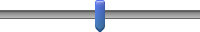 |
| --- | --- |
| **Moderate engagement:** for example, The genetic counselor **communicates directly with the moderator** of the Facebook group and can routinely check in to address supportive care needs **that the moderator requests** the genetic counselor’s input on. | 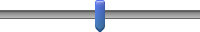 |
| **Enhanced moderate engagement:** for example, The genetic counselor **is a member** of the Facebook group and can post information, resources, and responses **directly to the group** as they see fit. | 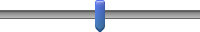 |
| **Maximum engagement:** for example, The genetic counselors **is a member** of the Facebook group and **can answer questions** from the group members. | 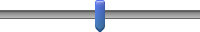 |

Q37 What do you think is the best way for genetic counselors to **engage with patients and/or their family members online**?

________________________________________________________________

Q38 Are you the **moderator** of the rare disease Facebook support group identified for this study?

Yes

No

The following questions ask about the **activities in the Facebook group where this survey was posted**.
 
Q39 Please rate each activity by its **frequency in your Facebook group** on a scale from 0 (never) to 10 (very frequently).

|  | never | | | | | | very frequently | | | | | |
| --- | --- | --- | --- | --- | --- | --- | --- | --- | --- | --- | --- | --- |
|  | 0 | 1 | 2 | 3 | 4 | 5 | | 6 | 7 | 8 | 9 | 10 |

| Members share or request information about **specialists** | 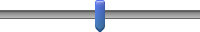 |
| --- | --- |
| Members share or request medical information about **symptoms or prognosis** | 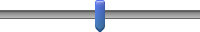 |
| Members share or request information about available **medications and other therapies** | 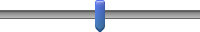 |

  Q40 Please rate each activity by its **frequency in your Facebook group** on a scale from 0 (never) to 10 (very frequently).

|  | never | | | | | | very frequently | | | | | |
| --- | --- | --- | --- | --- | --- | --- | --- | --- | --- | --- | --- | --- |
|  | 0 | 1 | 2 | 3 | 4 | 5 | | 6 | 7 | 8 | 9 | 10 |

| Members share or request information about **support services** (for example, respite care) | 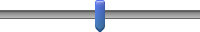 |
| --- | --- |
| Members share or request information about **events, articles, news, or educational opportunities** | 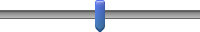 |
| Members share or request information about **research opportunities** | 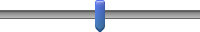 |

Q41 Please rate each activity by its **frequency in your Facebook group** on a scale from 0 (never) to 10 (very frequently).

|  | never | | | | | | very frequently | | | | | |
| --- | --- | --- | --- | --- | --- | --- | --- | --- | --- | --- | --- | --- |
|  | 0 | 1 | 2 | 3 | 4 | 5 | | 6 | 7 | 8 | 9 | 10 |

| Members share or request **social and emotional support** | 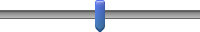 |
| --- | --- |
| Members discuss positive and negative **experiences** **with the healthcare system** | 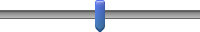 |
| Members discuss **physical challenges** of living with the rare disease | 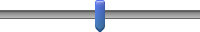 |
| Members discuss **financial concerns** and **healthcare costs** | 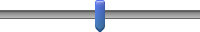 |

Q42 **Which of the following best describes your reason for engaging with** the Facebook group where this survey was posted?

I value the **social and emotional support** from other patients and families going through similar challenges.

I value the **practical information** I receive about managing my (my family member's) rare disease.

I value **both** the **social and emotional support** and the **practical information** equally.

Other (please specify):

Q43 If you had access to a genetic counselor through social media, what **type(s) of support would you request** from a genetic counselor?

The following questions will ask you about **your social media use**. To what extent **do you agree** with the following statements about the Facebook group where this survey was posted.

Q44 I feel **connected** to other people in this Facebook group.

strongly disagree

disagree

neutral

agree

strongly agree

Q45 I feel other Facebook group members **understand what I go through** every day.

strongly disagree

disagree

neutral

agree

strongly agree

Q46 I can talk to people in this Facebook group **about my day to day problems** if I need to.

strongly disagree

disagree

neutral

agree

strongly agree

Q47 What do you think are some **benefits** of engaging with genetic counselors online?

________________________________________________________________

Q48 What do you think are some **drawbacks** of engaging with genetic counselors online?

_______________________________________________________________
