# Supplementary material for: Perspectives of Rare Disease Social Media Group Participants on Engaging With Genetic Counselors: Mixed Methods Study
Source: J Med Internet Res. 2022 Dec 21;24(12):e42084. doi: 10.2196/42084 (PMC9813816; doi:10.2196/42084)
Supplement: Multimedia Appendix 2 [file jmir_v24i12e42084_app2.docx]

**Perspectives of Rare Disease Social Media Group Participants on Engaging with Genetic Counselors: Mixed Methods Survey**

Megan Yabumoto[1], Emily G. Miller[2], Anoushka Rao[2], Holly K. Tabor[2,3] Kelly E. Ormond[1,2,4], and Meghan C. Halley[2]

[1] Department of Genetics, Stanford University School of Medicine, Stanford, CA, USA

[2] Stanford Center for Biomedical Ethics, Stanford University School of Medicine, Stanford, CA, USA

[3] Department of Medicine, Stanford University School of Medicine, Stanford, CA, USA

[4] Health Ethics and Policy Lab, Department of Health Sciences and Technology, Swiss Federal Institute of Technology (Eidgenössische Technische Hochschule-Zurich), Zurich, Switzerland

**Supplemental Material 2: Supplemental Tables**

**Supplemental Table 1: Codebook for thematic analysis of open-ended questions**

| **Q1 – Logistics** | |
| --- | --- |
| One-on-one interactions | Any comment that explicitly states one-on-one interactions via telehealth (e.g. Zoom, Google Meet, video call), email, phone calls, or direct messaging. |
| Group-based interactions | Any comment referencing social media broadly, Facebook groups, website, forum, webinars, answering questions in a group setting as a helpful mode of communication. |
| Information sharing only | Any comment referencing posting information, announcements, educational materials without any one-on-one or group interactions. |
| Through moderator | Any comment referencing the moderator/other regulatory entity as the best way for a genetic counselor to interact with a support group. |
| Don’t bother | Any comment referring to the individual not wanting to engage in any form of these interactions via social media. |

| **Q2 – Information and Care Needs** | |
| --- | --- |
| Available to answer a questions | Any comment referencing the genetic counselor being able to answer questions about their disease (in real time, as they arise) |
| - Inquiries about family members | Any comment referencing the desire to understand the heritability of a disease (genetics, inheritance patterns), reproductive implications, and the impact a disease might have on the rest of their family. |
| - Inquiries about prognosis | Any comment referencing understanding what to expect in the future. |
| - Inquiries about treatment | Any comment referencing the need for updated information about potential treatments and practical support (management strategies, financial support). |
| Access to services | Any comment referencing the desire to gain access to genetic testing, specialists, others who might have more medical experience with their condition, and multidisciplinary teams. |
| Research/clinical trials | Any comment referencing the need for the most updated information about current research and the availability of clinical trials. |
| Unsure | Any comment explicitly stating “unsure” “not sure” “don’t know what a genetic counselor could help with” |

| **Q3 – Benefits** | |
| --- | --- |
| Accessible/convenient | Any comment that references better availability, ease of access due to lack of constraints around distance and time, and “quick and easy” access as a benefit. |
| Reliable information | Any comment that references gaining more information from personalized, vetted, reliable, trustworthy sources as a benefit. |
| Psychosocial support | Any comment that references the potential of a genetic counselor to provide support, faith, hope, and other coping mechanisms as a benefit. |
| No benefits | Any comment referring to the individual not wanting to engage in any form of these interactions via social media. |

| **Q4 – Drawbacks** | |
| --- | --- |
| Lack of personal relationship | Any comment referencing lack of having a direct, personal or pre-existing relationship as a drawback. |
| No drawbacks | Any comment explicitly stating that there are no perceived drawbacks to these interactions via social media. |
| Privacy/confidentiality | Any comment referencing concerns about privacy, security, and confidentiality as a drawback. |
| Lack of trust | Any comment referencing a lack of trust in the genetic counselor due to them not knowing their credentials, general mistrust of information, and biases GCs might have as a drawback. |
| Irrelevant information | Any comment referencing the genetic counselor providing information that is not pertinent to an individual’s certain circumstances (e.g., they provide information about a provider/service that is not available to them where they are located. |
| Frightening information | Any comment referencing an emotional response to the increased access to information they may/may not want to learn (e.g., fear, judgement, uncertainty). |
| Other | *Notes section – briefly summarize the comment* |

**Supplemental Table 2: Additional Individual Participant Level Characteristics (*N*=1035)**

|  | *n* | % |
| --- | --- | --- |
| **Type of community** | | |
| Rural area | 124 | 11.8% |
| Small city or town | 326 | 31.0% |
| Suburb near a large city | 338 | 32.1% |
| Large city | 236 | 22.4% |
| Missing | 29 | 2.8% |
| **Employment** | | |
| Not currently employed | 347 | 33.0% |
| Yes, part time | 160 | 15.2% |
| Yes, full time | 508 | 48.2% |
| Missing | 38 | 3.6% |
| **Household Size (**median, IQR) | | |
| 3.0 (2.0-4.0) | | |
| **Insurance ^b^** | | |
| Medicaid, CHIP, or other state-based program | 203 | 19.3% |
| Medicare | 139 | 13.2% |
| Private health insurance | 612 | 58.1% |
| Other health insurance | 131 | 12.4% |
| No health insurance | 87 | 8.3% |
| Missing | 44 | 4.2% |
| **Met with a genetic counselor** | | |
| Yes | 368 | 34.9% |
| No or don’t know/not sure | 573 | 54.4% |
| Missing | 112 | 10.6% |
| **Previous experience with genetic counselor(s) (*n* = 368)** | | |
| Extremely positive | 137 | 37.2% |
| Somewhat positive | 126 | 34.3% |
| Both positive and negative | 87 | 23.6% |
| Somewhat negative | 13 | 3.5% |
| Extremely negative | 5 | 1.4% |
| **Had genetic testing** | | |
| Yes | 539 | 51.2% |
| No or don’t know/not sure | 463 | 44.0% |
| Missing | 51 | 4.8% |
| **Experience with genetic counseling and testing** | | |
| Had genetic counseling AND genetic testing | 336 | 31.9% |
| Had genetic counseling but NO genetic testing | 32 | 3.1% |
| Had genetic testing but NO genetic counseling | 178 | 16.9% |
| Had NO genetic counseling and NO genetic testing | 395 | 37.5% |
| Incomplete data | 61 | 5.8% |
| Missing | 51 | 4.8% |
| **Obtained a genetic diagnosis through genetic testing** | | |
| Yes | 422 | 40.1% |
| Only a partial diagnosis OR only variants of uncertain significance | 69 | 6.6% |
| No known diagnosis (undiagnosed) | 429 | 40.7% |
| Other | 34 | 3.2% |
| Missing | 99 | 9.4% |
| **Type of support requested on social media** | | |
| Both social/emotional support and information on medical management | 489 | 46.4% |
| Information on medical management | 132 | 12.5% |
| Social/emotional support | 211 | 20.0% |
| Other | 11 | 1.0% |
| Missing | 210 | 19.9% |
| **I feel connected to other members** | | |
| Strongly disagree | 46 | 4.4% |
| Disagree | 39 | 3.7% |
| Neutral | 219 | 20.8% |
| Agree | 382 | 36.3% |
| Strongly agree | 149 | 14.2% |
| Missing | 218 | 20.7% |
| **Other members understand what I go through** | | |
| Strongly disagree | 31 | 2.9% |
| Disagree | 27 | 2.6% |
| Neutral | 104 | 9.9% |
| Agree | 395 | 37.5% |
| Strongly agree | 243 | 23.1% |
| Missing | 253 | 24.0% |
| **I can talk to others about what I go through** | | |
| Strongly disagree | 45 | 4.3% |
| Disagree | 73 | 6.9% |
| Neutral | 204 | 19.4% |
| Agree | 329 | 31.2% |
| Strongly agree | 164 | 15.6% |
| Missing | 238 | 22.6% |
| **Mean connectedness score (mean, standard deviation)** | | |
| 3.7 (0.8) | | |
| **I am interested in interacting with a genetic counselor on social media (median, IQR)** | | |
| 7.0 (4.0-9.0) | | |
| **Minimal Engagement (median, IQR)** | | |
| 5.0 (2.0-8.0) | | |
| **Moderate Engagement (median, IQR)** | | |
| 6.5 (4.5-8.5) | | |
| **Maximum Engagement (median, IQR)** | | |
| 9.0 (5.0-10.0) | | |
| **Disease Classification** | | |
| Rare skin disease | 140 | 13.3% |
| Rare developmental defect during embryogenesis | 132 | 12.5% |
| Rare neurologic disease | 124 | 11.7% |
| Rare gastroenterologic disease | 117 | 11.1% |
| Rare neoplastic disease | 107 | 10.2% |
| Rare inborn errors of metabolism | 88 | 8.4% |
| Rare endocrine disease | 79 | 7.5% |
| Rare hepatic disease | 69 | 6.6% |
| Rare bone disease | 50 | 4.7% |
| Rare systemic or rheumatologic disease | 44 | 4.2% |
| Rare renal disease | 37 | 3.5% |
| Rare ophthalmic disorder | 32 | 3.0% |
| Rare respiratory disease | 20 | 1.9% |
| Rare circulatory system disease | 6 | 0.6% |
| Rare hematologic disease | 3 | 0.3% |
| Rare immune disease | 3 | 0.3% |
| Rare cardiac disease | 2 | 0.2% |
| **Age of Onset^c^** | | |
| Antenatal | 103 | 9.8% |
| Neonatal | 238 | 22.6% |
| Infancy | 292 | 27.7% |
| Childhood | 389 | 36.9% |
| Adolescent | 524 | 49.8% |
| Adult | 523 | 49.7% |
| Elderly | 59 | 5.6% |
| All ages | 234 | 22.2% |
| Not available | 10 | 0.9% |
| **Inheritance Pattern^c^** | | |
| Autosomal recessive | 370 | 35.1% |
| Autosomal dominant | 347 | 33.0% |
| X-linked recessive | 48 | 4.6% |
| X-linked dominant | 52 | 4.9% |
| Multifactorial/multigenic | 13 | 1.2% |
| Mitochondrial inheritance | 6 | 0.6% |
| Oligogenic | 13 | 1.2% |
| Not available | 410 | 38.9% |
| **Facebook Group Privacy Settings** | | |
| Private | 1021 | 97.0% |
| Public | 32 | 3.0% |
| **Facebook Group Focus** | | |
| General | 838 | 79.6% |
| Parenting | 121 | 11.5% |
| Health Support | 69 | 6.5% |
| Social Learning | 25 | 2.4% |
| **Year Group was Created (median, IQR)** | | |
| 2010 (2008-2011) | | |

^a^Individuals can select more than one response.

^b^Information about the rare disease and social media group was integrated into the individual level data.

**Supplemental Table 3: Rare Disease and Social Media Group Level Characteristics (*N*=103)**

| **Rare Disease Social Media Support Groups Included (*n* = 103)** | ***n*** | **%** |
| --- | --- | --- |
| **Prevalence** | | |
| Unknown | 14 | 13.6% |
| <1 in 1,000,000 | 17 | 16.5% |
| 1-9 in 1,000,000 | 25 | 24.3% |
| 1-9 in 100,000 | 26 | 25.2% |
| 1-9 in 10,000 | 20 | 19.4% |
| >1 in 1,000 | 1 | 1.0% |
| **Disorder Type** | | |
| Disease | 85 | 82.5% |
| Malformation syndrome | 12 | 11.7% |
| Morphologic anomaly | 4 | 3.9% |
| Not available | 2 | 1.9% |
| **Disease Classification** | | |
| Rare neurologic disease | 19 | 18.4% |
| Rare developmental defect during embryogenesis | 16 | 15.5% |
| Rare inborn errors of metabolism | 13 | 12.6% |
| Rare skin disease | 12 | 11.7% |
| Rare neoplastic disease | 9 | 8.7% |
| Rare endocrine disease | 7 | 6.8% |
| Rare renal disease | 5 | 4.9% |
| Rare bone disease | 4 | 3.9% |
| Rare gastroenterologic disease | 3 | 2.9% |
| Rare hematologic disease | 3 | 2.9% |
| Rare ophthalmic disorder | 3 | 2.9% |
| Rare systemic or rheumatologic disease | 3 | 2.9% |
| Rare immune disease | 2 | 1.9% |
| Rare cardiac disease | 1 | 1.0% |
| Rare circulatory system disease | 1 | 1.0% |
| Rare hepatic disease | 1 | 1.0% |
| Rare respiratory disease | 1 | 1.0% |
| **Age of Onset^a^** | | |
| Antenatal | 12 | 11.7% |
| Neonatal | 32 | 31.1% |
| Infancy | 34 | 33.0% |
| Childhood | 32 | 31.1% |
| Adolescent | 24 | 23.3% |
| Adult | 36 | 35.0% |
| Elderly | 7 | 6.8% |
| All ages | 21 | 20.4% |
| Not available | 4 | 3.9% |
| **Inheritance Pattern^a^** | | |
| Autosomal recessive | 38 | 36.9% |
| Autosomal dominant | 33 | 32.0% |
| X-linked recessive | 9 | 8.7% |
| X-linked dominant | 3 | 2.9% |
| Multifactorial/multigenic | 9 | 8.7% |
| Mitochondrial inheritance | 2 | 1.9% |
| Oligogenic | 1 | 1.0% |
| Not available | 32 | 31.1% |
| **Number of groups contacted** | | |
| 425 | | |
| **Total number of groups who responded** | | |
| 110 | | |
| **Total number of groups meeting inclusion criteria** | | |
| 103 | | |
| **Moderator of Facebook group** | | |
| Yes | 109 | 10.4% |
| No | 770 | 73.1% |
| Missing | 174 | 16.5% |
| **Facebook Group Disease Specification** | | |
| Specific to rare disease | 94 | 91.3% |
| “Umbrella” rare disease | 9 | 8.7% |
| **Year Group was Created (median, IQR)** | | |
| 2012 (2010-2015) | | |
| **Privacy Settings** | | |
| Private | 97 | 94.2% |
| Public | 6 | 5.8% |
| **Group Focus** | | |
| General | 83 | 80.6% |
| Parenting | 10 | 9.7% |
| Health Support | 8 | 7.8% |
| Social Learning | 2 | 1.9% |
| **Size of Group (median, IQR)** | | |
| 634 (143.5-1850.0) | | |
| **Number of new posts per month (median, IQR)** | | |
| 20 (3.0-64.5) | | |
| **Number of new members per week (median, IQR)** | | |
| 2 (0.0-6.0) | | |

^a^There was more than one response listed.

**Supplemental Table 4: Interest in Engaging with Genetic Counselors by Variable**

| **Interest in Engaging with Genetic Counselors** | | | | |
| --- | --- | --- | --- | --- |
| **Variable** | **Mean 1** | **Mean 2** | ***t* statistic** | ***p*-value** |
| Patient (vs family member) | 6.34 | 6.18 | 0.68 | 0.50 |
| Moderator (vs not a moderator) | 5.90 | 6.38 | 1.35 | 0.18 |
| Met with a GC (vs did not meet with a GC) | 6.01 | 6.47 | 2.09 | 0.04* |
| Had genetic testing (vs did not have genetic testing) | 6.16 | 6.44 | 1.29 | 0.20 |
| Values informational support (vs not) | 6.32 | 6.43 | 0.45 | 0.65 |
| Values social and emotional support (vs not) | 6.40 | 6.10 | -0.93 | 0.35 |
| Above mean connectedness score (vs below) | 6.39 | 6.33 | -0.26 | 0.80 |
| Lives in rural community (vs not) | 6.58 | 6.25 | -0.64 | 0.52 |
| Lives in suburb near large city (vs not) | 6.23 | 6.30 | 0.28 | 0.78 |
| Lives in small city or town (vs not) | 6.10 | 6.36 | 1.08 | 0.28 |
| Lives in large city (vs not) | 6.49 | 6.22 | -1.03 | 0.31 |
| Less than bachelor’s degree (vs bachelor’s degree and above) | 6.20 | 6.44 | 1.08 | 0.28 |
| Ultra-rare prevalence (vs not) | 6.35 | 6.26 | 0.34 | 0.73 |
| Above average Facebook group size (vs below) | 6.46 | 6.17 | -1.30 | 0.20 |
| **Variable** | **Sum of squares (*df*)** | **Mean square** | ***F* statistic** | ***p*-value** |
| Income below $50K vs Income $50-100K vs Income above $100K | 25 (1) | 24.57 | 2.50 | 0.12 |

****p<0.001, **p<0.01, *p<0.05*
